# Extended weekly dose-dense paclitaxel/carboplatin is feasible and active in heavily pre-treated platinum-resistant recurrent ovarian cancer

**DOI:** 10.1038/sj.bjc.6604914

**Published:** 2009-02-17

**Authors:** R Sharma, J Graham, H Mitchell, A Brooks, S Blagden, H Gabra

**Affiliations:** 1 Ovarian Cancer Action (HHMT) Research Centre, Imperial College London Hammersmith Campus, Du Cane Road, London W12 ONN, UK; 2 Imperial College Healthcare Trust, Charing Cross Hospital, Fulham Palace Road, London W6 8RF, UK

**Keywords:** ovarian cancer, resistance, relapse, carboplatin, paclitaxel, weekly

## Abstract

There is increasing evidence of the efficacy of dose-dense therapy in the management of platinum-resistant/refractory ovarian cancer. We report our experience of extended weekly carboplatin and paclitaxel in this population group. Twenty patients with platinum-resistant/refractory ovarian cancer received carboplatin AUC 3 and paclitaxel 70 mg m^−2^ on day 1, 8, 15 q 4 weekly for six planned cycles. Toxicity was assessed using Common Toxicity Criteria. Response was evaluated using radiological and CA125 criteria. Median age was 61 years (range 40–74 years). Median number of prior therapies is three (range 1–8). Response rate was 60% by radiological criteria (RECIST) and 76% by CA125 assessment. Grade 3 toxicities consisted of neutropenia (29% of patients) and anaemia (5%). One patient experienced grade 4 neutropenia. No grade 3/4 thombocytopaenia was reported. Fatigue, nausea and peripheral neuropathy were the most frequent non-hematological side effects. Median progression-free survival was 7.9 months and overall survival was 13.3 months. The dynamics of response to dose-dense therapy were as rapid as with front-line therapy within the same patient. This dose-dense regimen can be extended to at least 18 weekly cycles over 6 months and is well tolerated with high response rates in heavily pre-treated, platinum-resistant ovarian cancer. It forms a highly active and tolerable cytotoxic scaffold to which molecular-targeted therapies can be added in platinum-resistant ovarian cancer.

Ovarian cancer is the leading cause of death from gynecologic malignancies in the United Kingdom, and is the fourth most common cause of cancer mortality in women (Piver *et al*, 1991). Despite relatively high response rates to first-line platinum-based therapies, the majority of patients with epithelial ovarian cancer will experience disease relapse and will require further chemotherapy (Neijt *et al*, 1991). Several therapeutic options are available and the decision as to which therapy to commence is dependent on the time from last platinum chemotherapy to decision to treat, known as the platinum-free interval (PFI) (Markman *et al*, 1991; van der Burg *et al*, 1991). The PFI is a predictor of response not only to second-line treatment with platinum-based chemotherapy but also other active agents including the taxanes, topoisomerase I inhibitors and anthracyclines (Eisenhauer *et al*, 1994). The probability of response to platinum rechallenge increases with the PFI, from >60% in patients relapsing >12 months since last platinum therapy to below 10% in patients relapsing within 6 months (Markman *et al*, 1991). This entity of platinum-resistant ovarian cancer, therefore, represents a different clinical scenario with lower response rates between 10 and 15% to a range of chemotherapy agents (Gordon *et al*, 2004; Mutch *et al*, 2007; Ferrandina *et al*, 2008). However, there is increasing evidence that by administering platinum in a ‘dose-dense’ manner involving increased schedule frequency and increased dose intensity, resistance can be overcome resulting in significant improvements in response (van der Burg *et al*, 2005).

Thus, dose density refers to increasing dose frequency while the dose per cycle and overall dose remains the same, thereby increasing the dose intensity and shortening overall treatment time (Simon and Norton, 2006). A number of different ‘dose-dense’ carboplatin and paclitaxel regimens have been developed for the treatment of recurrent ovarian cancer initially pioneered by van der Burg *et al* (2002). They reported a response rate of 46% in patients with a PFI of <4 months with weekly cisplatin (50–70 mg m^−2^) and daily oral etoposide for 6 weekly cycles, followed by maintenance oral etoposide. On the basis of finding that weekly paclitaxel is also very active in platinum-resistant disease, cisplatin and carboplatin have both been tolerably combined with paclitaxel in short duration weekly schedules. de Jongh *et al* (2002) and Van der Burg *et al* (2004) have reported on weekly induction regimens of cisplatin 70 mg m^−2^ or carboplatin AUC 4 with paclitaxel 90 mg m^−2^ for 6 weeks, followed by a return to three weekly schedules. The authors report a response rate of 60% in platinum-resistant disease and progression-free survival of 8–11 months. However, if the dose-dense regimen is likely to be the more active component of this regimen, there is a clear rationale for continuing with weekly treatment rather than reverting to a three weekly ‘maintenance’ schedule to which patients had prior resistance. In practice, with these Rotterdam regimens, the limitation in the dose-dense phase was toxicity.

Weekly dosing was also investigated earlier as part of the ‘Leuven’ dose regimen in which patients received continuous weekly carboplatin AUC 4 and paclitaxel 90 mg m^−2^ on day 1 and 8 every 21days for 6 cycles (Cadron *et al*, 2007). The authors reported a response rate of 38% in platinum-resistant patients with a progression-free survival of 6.8 months and an overall survival of 8 months; these results were achieved with significant haematological toxicity. Havrilesky *et al* (2003) investigated weekly carboplatin AUC 2 and paclitaxel 80 mg m^−2^ on day 1, 8 and 15 every 28 days until disease progression; thus not delivering dose-dense platinum, as the platinum dose intensity in this regimen is below that achieved with conventional schedules. For this regimen, a median of 5.5 cycles (477 pulses) was administered with a similar response rate of 38% reported in platinum-resistant patients and a progression-free survival of 3.2 months, and an overall survival of 11 months. Although these studies suggest that weekly platinum and taxane is an effective and well-tolerated approach in recurrent ovarian cancer, neither the optimal schedule nor its duration has been determined. This is important from the perspective not just of response rate and maximal tolerated dose but also from the perspective of potential durability of response in platinum-resistant disease.

Here, we report our experience of dose dense weekly carboplatin AUC 3 and paclitaxel 70 mg m^−2^ on day 1, 8, 15 q 4 weekly administered in patients with heavily pre-treated platinum-refractory and/or platinum-resistant ovarian cancer delivered for an intended six blocks of therapy (18 cycles). It shows excellent feasibility, tolerability and activity of this regime in a very difficult patient group. The importance and novelty of this regime is its feasibility and tolerability, and its potential for extended duration therapy allows us to contemplate its use as a scaffold for the safe integration of targeted therapies that may enhance the efficacy of platinum rechallenge in a platinum-resistant scenario.

## Methods

This is a retrospective report of our experience of patients with a diagnosis of epithelial ovarian cancer who had relapsed within a platinum-resistant interval (⩽6 months) treated with dose-dense carboplatin and paclitaxel. Carboplatin AUC 3 and paclitaxel 70 mg m^−2^ were administered on day 1, 8, 15 q 4 weekly, with the intent to deliver six cycles of chemotherapy. Baseline CT imaging of the chest, abdomen and pelvis was carried out prior to the commencement of therapy and after every two cycles. Carboplatin dose was calculated by ethylenediaminetetraacetic acid clearance. Blood samples for full blood count, biochemistry, liver function tests and serum CA125 test were taken prior to the commencement of therapy and before each treatment. Patients were reviewed weekly during treatment for safety assessment. All safety evaluations were graded according to the NCI-CTC v5.0. Tumour response was assessed after every two cycles with repeat CT chest, abdomen and pelvis (RECIST criteria) and by CA125 (GCIG criteria) (Therasse *et al*, 2000; Rustin, 2003). Data regarding the planned and delivered weekly dose intensity of treatment, the overall treatment dose delivered, toxicity and clinical outcome were collected.

### Survival

Progression-free survival was measured from the date of first treatment until either progression or death, the median OS was defined as the period of time between the start of the treatment and death and was censored at last follow-up. Survival curves were generated using Kaplan–Meier methodology. Univariate survival analyses were performed using the Kaplan–Meier method and log rank tests. The relationship between subgroup variability and response to treatment was analysed using either *χ*^2^-test or Fisher's exact test. Analysis was performed using SPSS software version 11.5 (SPSS Inc., Chicago, IL, USA).

## Results

### Patients

Twenty-three patients received dose-dense carboplatin/paclitaxel between August 2006 and August 2008. Two patients were excluded from analysis as one initially received cisplatin/etoposide and one received two cycles of three weekly carboplatin immediately prior to dose-dense therapy. All patients had histologically proven epithelial ovarian cancer. All patients had been treated earlier with carboplatin, and 18 (86%) patients had previous conventional schedule taxane exposure. Patient characteristics are shown in Table 1. The median age of the evaluable patients was 61 years (range 40–74 years). The majority of patients had a performance status of one (71%). Patients had received an average of three (range 1–8) prior chemotherapeutic regimens. Seven patients had platinum refractory disease following front-line therapy. The mean PFI was 5.1 months (range 0.5–13.9 months). The mean taxane-free interval was 16.8 months (range 1.2–75.1 months). Three patients had no prior exposure to taxanes. All patients had relapsed within a platinum-resistant time period and median time to relapse following platinum-based therapy is 5.4 months (range 0–6.7). However, three patients received treatment with non-platinum conventional chemotherapy regimens (two received liposomal doxorubicin and one received topotecan). Six patients symptomatically relapsed but were not treated within 6 months either because of prolonged hospitalisation or because patients elected to delay treatment. The mean treatment-free interval was 4.7 months (range 0.5–12.1 months).

An average of four blocks of dose-dense chemotherapy was administered (range 1–6). The mean delivered dose intensity of carboplatin per week was AUC 2.1 (range 0.5–2.25) against a planned dose intensity of AUC 2.25. The median ratio of carboplatin delivered *vs* planned dose was 95% (range 23–100). Although no dose reduction in paclitaxel dosing was required, a reduction in dose intensity was observed that was attributable to dose delay. The mean delivered dose intensity of paclitaxel was 50 mg m^−2^ per week (range 11.8–52.5) against a planned dose intensity of 52.5 mg m^−2^ per week. The median ratio of paclitaxel delivered *vs* planned dose was 91% (range 22–100).

### Treatment efficacy

One patient was not assessed radiologically because of death prior to assessment. Therefore, 20 patients were assessed for radiological response. Confirmed partial tumour responses (RECIST) were reported in 12 patients (60%). No complete responses were reported. Four patients (20%) had stable disease and four (20%) patients had progressive disease on treatment. All patients were assessable for response by GCIG CA125 criteria. In all, 16 patients (76%) achieved a response (Table 2).

The rate of decline in CA125 was assessed in 18 patients compared with their rate of CA125 decline with first-line therapy. Three patients commenced their first-line treatment in other institutions and therefore their initial serum CA125 results were not available. In patients receiving dose-dense therapy the median half-life of serum CA125 was 16.5 days (range 0–41). The median half-life of serum CA125 in this population at the time of front-line treatment was longer at 18 days (range 0–42) (Table 3). Analysis of patients whose CA125 half-life response to dose-dense therapy was faster compared with front-line therapy revealed longer overall survival (mean overall survival 20.2 months (95% CI 12.5–27.9) *vs* 8.8 months (95% CI 6.1–11.6), *P*=0.018) but no difference in progression-free survival (PFS). No association was observed between CA125 half-life response to dose-dense therapy and PFI or exposure to prior taxane therapy.

**Figure 1:**
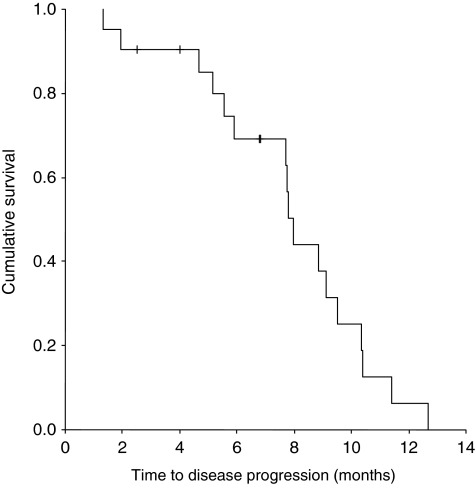
Kaplan–Meier curve for time to disease progression in patients receiving dose-dense carboplatin and paclitaxel for platinum-resistant ovarian cancer.

**Figure 2:**
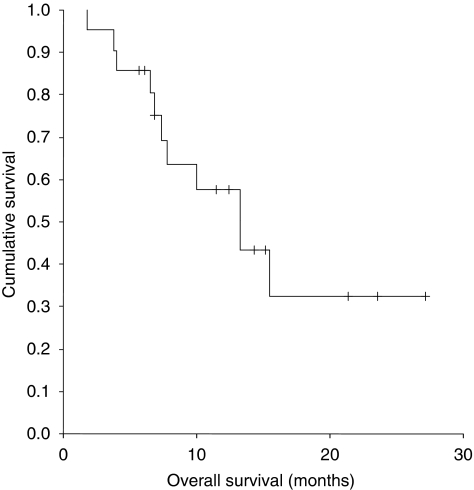
Kaplan–Meier curve for overall survival patients receiving dose-dense carboplatin and paclitaxel for platinum-resistant ovarian cancer.

In the 16 patients who had been exposed to a taxane prior to weekly carboplatin/paclitaxel, the CA125 response rate was 81% and the radiologic response rate was 83%. There was no correlation between the response rate to dose-dense therapy, by either RECIST or GCIG criteria, and platinum-free interval, taxane-free interval or treatment-free interval. At the time of analysis, 11 patients had died. The median PFS was 7.9 months (95% CI 7.59–8.35) (Figure 1). The median overall survival was 13.3 months (95% CI 7.81–18.79) (Figure 2).

### Toxicity

Overall dose-dense carboplatin/paclitaxel was well tolerated (Table 4). Six patients (29%) experienced grade 3 neutropenia and one patient experienced grade 4 neutropenia. Two patients required admission for neutropenic sepsis (10%). One patient (5%) died as a result of sepsis. One patient experienced non-neutropenic line sepsis (5%). One patient (5%) experienced symptomatic grade 3 anaemia. Importantly, grade 3/4 thrombocytopenia or grade 4 anaemia were not observed.

Three patients required dose reductions secondary to neutropenia (14%), and one patient required two additional dose reductions because of persistent neutropenia. Eight patients required dose delays (38%), predominantly because of neutropenia; however, we dose delayed patients rather than administer granulocyte-colony stimulating factor (G-CSF). Only three patients received G-CSF therapy (14%). G-CSF was administered 300 *μ*g subcutaneously daily from day 3 to day 5. Three patients (14%) experienced treatment delays because of Unit holidays.

The most common non-haematologic toxicity was grade 2 lethargy (33%). Two patients (10%) experienced grade 2 nausea and vomiting. Three patients developed worsening of pre-existing peripheral neuropathy leading to the cessation of treatment. Three patients (14%) developed hypersensitivity reaction secondary to carboplatin infusion. One patient was managed effectively with pre-treatment with hydrocortisone 100 mg intravenously and chlorphenamine 10 mg intravenously prior to carboplatin infusion. Two patients ceased therapy because of hypersensitivity reactions to carboplatin.

## Discussion

Several chemotherapeutic agents such as topotecan, gemcitabine, liposomal doxorubicin, paclitaxel and etoposide have been used in the treatment of platinum-resistant disease with unexciting response rates in the range 10–15% in this patient group (Swisher *et al*, 1997; Gordon *et al*, 2001). Our data show that the use of extended dose-dense chemotherapy results in a response rate of 60% in this poor prognosis group, which is consistent with previous published studies (de Jongh *et al*, 2002; Van der Burg *et al*, 2004). Extended dose-dense therapy affords the opportunity to effectively and tolerably treat conventionally platinum-resistant patients with platinum again, and this study shows the feasibility and tolerability of delivering up to six blocks (18 cycles) of this treatment. This finding is of importance as platinum resistance ultimately becomes the dominant problem for most patients with ovarian cancer. The approach described here holds promise in that it may be coupled to molecularly targeted therapies that can either reverse acquired platinum resistance or enhance intrinsic platinum sensitivity (Gordon *et al*, 2005; Schilder *et al*, 2005; Alvero *et al*, 2006; Appleton *et al*, 2007; Cannistra *et al*, 2007; Chien *et al*, 2007). This approach may feasibly enhance efficacy to the point that survival gains may become worthwhile. The regimen described in this report, therefore, needs to be tested in a phase III setting against the community standard for this population, pegylated liposomal doxorubicin.

The mechanism by which dose-dense therapy induces responses in patients with platinum-resistant disease is unclear. It is assumed that a fixed proportional cell kill is achieved at shorter time intervals, improving the overall impact of therapy, as it allows less time and opportunity for the emergence and proliferation of surviving cells. Furthermore, for S phase-specific drugs, more frequent dosing may expose more cells to the drug during the sensitive phase of the cell cycle. In addition, the use of weekly paclitaxel may have additional anti-angiogenic effects when used in a fractionated schedule (Belotti *et al*, 1996; Lau *et al*, 1999).

As outlined in the introduction, dose dense weekly schedules of carboplatin and paclitaxel have been investigated by a number of groups. These studies all differ in the dose intensity of carboplatin and paclitaxel administered, the duration of dose-dense therapy and the concurrent toxicity (Bolanos *et al*, 2001; Katsumata *et al*, 2001; Dunton, 2003; Havrilesky *et al*, 2003; Van der Burg *et al*, 2004; Kikuchi *et al*, 2005; Rose *et al*, 2005; Watanabe *et al*, 2005; Cadron *et al*, 2007). However, only four of the nine identified published studies enrolled patients with platinum-resistant disease (Table 5). The results from our study (a response rate of 60% and a PFS of 7.9 months in resistant or refractory disease) are comparable with these previously reported data, although the extended duration to six blocks (12 cycles total) has only been shown in the Leuven study, which with an AUC 4/taxol 90 mg m^−2^ dosing could be delivered only as 2 weeks on and 1 week off, with significant haematological toxicity, which therefore could make integration of additional biotherapies difficult (Cadron *et al*, 2007).

We calculated the half-life of serum CA125, and have shown a more rapid decline in CA125 after dose-dense therapy in platinum-resistant disease compared with CA125 decline in front-line therapy for individual patients. Furthermore, we have shown that patients whose CA125 half-life response to dose-dense therapy was faster compared with front-line therapy had an improved overall survival when compared with those with a slower response. This is consistent with previously published front-line studies, which suggest that a shorter half-life is associated with improved prognosis (Riedinger *et al*, 2008) (Mano *et al*, 2005) (Gadducci *et al*, 2004). No study, however, has compared the decline in CA125 within patients with subsequent treatments and the impact on clinical outcomes. Although interesting, these results are based on a small sample size and need confirmation in a larger patient population.

Although all the patients relapsed within a platinum-resistant interval, treatment was delayed in nine patients such that these patients did not receive a platinum regimen within 6 months. Despite this, no differences were observed in response rates for this group; however, it is possible that this subgroup may have been more sensitive (resensitised) to platinum. A significant minority of patients were not prior taxane exposed, which may also have affected response to weekly therapy, although the small sample size for this subgroup precluded statistical evidence for this. Nevertheless, this is overall one of the largest reports of dose dense weekly therapy in the resistant patient population and further supports this approach to treatment in a difficult clinical scenario. The ‘3 weeks on, 1 week off’ regimen described here differs from those published earlier in its tolerability and, therefore, potential for extension of therapy for at least 18 cycles, over 24 weeks and possible use for treatment to progression (which is not a standard approach in the United Kingdom, but may be more acceptable in other countries).

We have shown that dose-dense carboplatin/paclitaxel regimen can be administered safely with minimal toxicity in heavily pre-treated platinum-resistant patients. In particular, we report a negligible incidence of thrombocytopenia, the most important and unaddressable dose limiting factor in this approach, and a low incidence of grade 3/4 neutropenia (30%) in contrast to other published studies. In particular, the study by Cadron *et al* (2007) investigating carboplatin AUC 4 and paclitaxel 90 mg m^−2^ on day 1 and 8 every q 4 weekly reported an incidence of grade 3/4 neutropenia in 94% and thrombocytopenia in 25% of patients. These toxicities would have curtailed the extended duration of the use of this regimen and feasibility for integration with other molecularly targeted therapies. Only one previous study examining carboplatin AUC 2 and paclitaxel 80 mg m^−2^ reports a comparable rate of neutropenia; however, the authors report a higher incidence of grade 3/4 thrombocytopenia, and this study does not deliver dose-dense platinum, indeed the dose intensity of platinum is less than that of conventional therapy (Havrilesky *et al*, 2003). Furthermore, we report a low incidence of anaemia compared with the previous reports. We did observe a significant number of dose delays because of neutropenia. As described above, we tended to dose delay rather than administer G-CSF in myelosuppression and this may have compromised the dose intensity of delivered chemotherapy. In future studies, we propose the addition of G-CSF 300 *μ*g daily from day 3 to 5 to avoid neutropenia and maintain dose intensity, and it will be interesting to observe whether this will impact thrombocytopenia adversely. Interestingly, we also did not observe a high rate of neurotoxicity despite the majority of patients having had prior treatment with a taxane. Because of the impact of neurotoxicity on patient quality of life, we plan to formally assess neuropathy prior to and post dose-dense therapy in future studies. A major limitation to the use of dose-dense therapy is the development of hypersensitivity to platinum. Previous studies do report hypersensitivity occurring in 6–23% of patients treated. We report 14% of patients developing hypersensitivity. Although one patient was effectively managed with the use of hydrocortisone and anti-histamines, two patients had to cease treatment. Desensitisation to platinum agents is an active area of research and this may impact favourably on the deliverability of platinum agents in the future.

This regimen is therefore a safe and effective regimen for the management of platinum-resistant relapse, and efforts are underway to explore this regimen in randomised clinical trials against the current standard of care in platinum-resistant ovarian cancer. Translational research efforts are also showing that inhibition of several molecular targets can reverse acquired clinical platinum resistance *in vitro*, and adaptations of this regimen will be explored as a platinum scaffold to incorporate inhibitors to such targets in order to maximise clinical benefit to patients with platinum-resistant ovarian cancer (Stronach *et al*, 2008).

## Figures and Tables

**Table 1:** Patient demographics (*N*=21)

**Patient demographics**	**No of patients (%)**
*Age, years*	
Median	62
Range	41–75
	
*WHO performance status*	
0	6 (29)
1	15 (71)
2	0
	
*Tumour histology*	
Serous	19 (90)
Endometrioid	2 (10)
Mucinous	0
Clear cell	0
	
*Tumour differentiation grade*	
1	0
2	4 (20)
3	16 (76)
Unknown	1 (5)
	
*Number of prior chemotherapeutic regimens*	
1	7 (33)
2	6 (29)
3	5 (24)
⩾4	3 (14)
	
*Time to relapse following last chemotherapy*	
Median	0.7 months
Range	0–6.7
	
*Platinum-free interval, months*	
<6	12
⩾6	9^a^
	
*Taxane-free interval, months*	
<6	8
⩾6	11
No prior taxane	3

a Six patients relapsed within a platinum-resistant time period (<6 months) but elected to delay treatment. Three patients received alternative chemotherapy for the treatment of platinum-resistant disease.

**Table 2:** Overall response rates to dose-dense carboplatin/paclitaxel

**Response type**	**No. of patients (%)**
	**Radiologic response**
Complete response	0
Partial response	12 (60)
Stable disease	4 (20)
Progressive disease	4 (20)

**Table 3:** Half-life of serum CA125 (days) during front-line chemotherapy treatment and subsequent half-life with dose-dense chemotherapy

**Subject number**	**First-line chemotherapy: half-life CA125 (days)**	**Dose-dense therapy: half-life CA125 (days)**
1	21	30
2	17	11
3	7.3	15
4	24	21
5	12	10
6	33	14
7	30	41
8	—	18
9	—	R
10	37	R
11	20	33
12	13	R
13	R	37
14	—	30
15	12	12
16	17	23
17	42	35
18	10	10
19	19	23
20	12	28
21	19	18
Median	18	18

R=Refractory.

**Table 4:** Haematologic and non-haematologic toxicity of dose-dense therapy

**Grade no (%)**	**1**	**2**	**3**	**4**
Anaemia	2 (10)	13 (62)	1 (5)	0
Neutropenia	4 (19)	5 (24)	6 (29)	1 (5)
Thrombocytopaenia	1 (5)	1 (5)	0	0
Lethargy	3 (14)	8 (38)	0	0
Nausea and vomiting	4 (19)	2 (10)	0	0
Oral mucositis	2 (10)	0	0	0
Arthralgia	1 (5)	1 (5)	0	0
Neurotoxicity	4 (19)	1 (5)	3 (14)	0
Renal toxicity	1 (5)	1 (5)	0	0
Otoxicity	1 (5)	1 (5)	0	0

**Table 5:** Studies of weekly carboplatin and paclitaxel in platinum-resistant recurrent ovarian cancer

**Study**	**Regimen**	**Platinum sensitivity**	***N***	**RR (%)**	**CR (%)**	**PFS (months)**	**OS (months)**	**Anaemia**	**Neutropaenia**	**Thrombocytopaenia**	**Neurotoxicity**	**Alopecia**	**Hypersensitivity**
Cadron *et al* (2007)	T 90 mg m^−2^ per week	Total number	29	66	21	9	18	24	94	25	3	0	9
	C AUC 4 per week	<6 months	10	38	13	6.8	8						
	Days 1, 8, q 3 weekly	6–12 months	11	73	18	10.5	NR						
	6 courses	>12 months	8	80	30	12.8	NR						
						2.6							
Van der Burg *et al* (2004)	T 90 mg m^−2^ per week	Total number	62	74	24	11	NR		40	8	0		23
	C AUC 4 per week	<6 months	23	61	13								
	Days 1, 8, 15, 29, 36, 49	6–12 months	19	84	42								
	+6xTC q 3 weekly	>12 months	20	80	20								
Havrilesky *et al* (2003)	T 80 mg m^−2^ per week	Total number	29	83	55	11.5		11	32	14	0	0	21
	C AUC 2 per week	<6 months	8	38	13	3.2	11.4						
	Days 1, 8, 15 q 28 days until progression/CR+8 courses	>6 months	21	100	71	13.7							
Katsumata *et al* (2001)	T 80 mg m^−2^ per week	Total number	45 (33)	67				39	61	15	21		6
	C AUC 2 per week	<6 months	11	55							(+ grade 2)		
	for 18 weeks	>6 months	16	73									
Current study	T 70 mg m^−2^ per week	Total number	21	60	0	7.9	13.3	5	30	0	15	NR	14
	C AUC 3 per week	Refractory	5	25		8.9	6.8						
	Days 1, 8, 15 q 4 weekly	<6 months	7	25		5.5	—						
	4–6 courses	>6 months	9	50		7.8	10.0						

*N*=number of patients; RR=response rate; CR=complete remission; PFS=progression-free survival; OS=overall survival; T=paclitaxel; C=carboplatin; NR=not reached.

Toxicity is grade ⩾3 and expressed in percentage. Adapted from Cadron *et al* (2007).
